# Data set for the proteomics analysis of the endomembrane system from the unicellular *Entamoeba histolytica*

**DOI:** 10.1016/j.dib.2014.08.007

**Published:** 2014-09-03

**Authors:** Doranda Perdomo, Nawel Aït-Ammar, Sylvie Syan, Martin Sachse, Gagan Deep Jhingan, Nancy Guillén

**Affiliations:** a Institut Pasteur, Cell Biology of Parasitism Unit, F-75015 Paris, France; b INSERM U786, F-75015 Paris, France; c Université Paris Diderot, Sorbonne Paris Cité, Cellule Pasteur, F-75015 Paris, France; d Institut Pasteur, Imagopole, Plate-Forme de Microscopie Ultrastructurale, France; e National Institute of Immunology, Aruna Asaf Ali Marg, New Delhi, India

## Abstract

*Entamoeba histolytica* is the protozoan parasite agent of amebiasis, an infectious disease of the human intestine and liver. This parasite contact and kills human cells by an active process involving pathogenic factors. Cellular traffic and secretion activities are poorly characterized in *E. histolytica*. In this work, we took advantage of a wide proteomic analysis to search for principal components of the endomembrane system in *E. histolytica*. A total of 5683 peptides matching with 1531 proteins (FDR of 1%) were identified which corresponds to roughly 20% of the total amebic proteome. Bioinformatics investigations searching for domain homologies (Smart and InterProScan programs) and functional descriptions (KEGG and GO terms) allowed this data to be organized into distinct categories. This data represents the first in-depth proteomics analysis of subcellular compartments in *E. histolytica* and allows a detailed map of vesicle traffic components in an ancient single-cell organism that lacks a stereotypical ER and Golgi apparatus to be established. The data are related to [1].

Data are supplied here and have been deposited to the open access library of ProteomeXchange Consortium (http://www.proteomexchange.org) via the PRIDE partner repository [2] with the dataset identifier PXD000770

**Table:** **Specificationstable**

Subject area	Biology, parasitology
More specific subject area	Proteomics on the endomembrane system of *Entamoeba histolytica*
Type of data	Proteome Discoverer and Maxquant results (.txt) and list of identified proteins as tables (.xls)
How data was acquired	Liquid chromatography mass spectrometry in tandem (LC–MS/MS). Proteins from the internal membrane fraction of *E. histolytica* trophozoites were treated to obtain tryptic peptides. These were separated by HPLC coupled to an LTQ-Orbitrap Velos mass spectrometer (Thermo Fisher Scientific)
Data format	Raw and analyzed
Experimental factors	Non applied
Experimental features	Cell fractionation of *E. histolytica* to obtain enriched endomembrane proteins as described before [4] with some modifications. Samples were then prepared for liquid chromatography–mass spectrometry (LC–MS/MS) analysis. (Fig. 1)
Data source location	Paris, France. Institut Pasteur.
Data accessibility	Data are supplied here and have also been deposited to the open access library of ProteomeXchange Consortium (http://www.proteomexchange.org) via the PRIDE partner repository [2] with the dataset identifier PXD000770

**Value of the data**

• First in-depth proteomics analysis of subcellular compartments in *E. histolytic*.
• Proteomics characterization of the endomembrane network in *E. histolytica*.
• Strong iBAQ intensity values from the protein spectra indicative of abundance and relevant to the construction of amebic intracellular trafficking components.

## 1 Data, experimental design, materials and methods

### 1.1 Preparation of samples for proteomics analysis

Proteins from the internal membrane fraction (50 µg) were precipitated with the methanol–chloroform method [3] and the resulting dried pellet was dissolved in freshly prepared digestion buffer (8 M urea in 25 mM NH_4_HCO_3_). Sample were reduced with 5 mM TCEP (45 min, 37 °C) and alkylated with 50 mm iodoacetamide (60 min, 37 °C) in the dark. Sample were diluted with 25 mM NH_4_HCO_3_ to a final concentration of 1 M urea and digested overnight at 37 °C with sequencing grade trypsin gold (1 µg, Promega USA). After digestion, peptide mixtures were acidified to pH 2.8 with formic acid and desalted with minispin C18 columns (Nestgrp, USA). Samples were dried under vacuum and solubilized in 0.1% formic acid and 2% acetonitrile before mass spectrometric analysis.

### 1.2 Liquid chromatography–mass spectrometry (LC–MS/MS) analysis of proteins

The tryptic peptide samples (1 µl roughly containing 1 µg) were separated by reverse-phase chromatography for each experiment via Thermo Scientific Proxeon nano LC using a C18 picofrit analytical column (360 μm OD, 75 μm ID, 10 μm tip, Magic C18 resin, 5 µm size, Newobjective, USA). The HPLC was coupled to an LTQ-Orbitrap Velos mass spectrometer (Thermo Fisher Scientific). Peptides were loaded onto the column with Buffer A (2% acetonitrile, 0.1% formic acid) and eluted with 120 min linear gradient from 2 to 40% buffer B (80% acetonitrile, 0.1% formic acid). After the gradient the column was washed with 90% buffer B and finally equilibrated with buffer A for next run. The mass spectra were acquired in the LTQ Orbitrap velos with full MS scan (RP 30,000) followed by 10 data-dependent MS/MS scans with detection of the fragment ions in the FTMS HCD mode (RP 7500). Target values were 1×10^6^ for full FT-MS scans and 5×10^4^ for FT-MS MS*n* scans. Ion selection threshold was set to 5000 counts.

### 1.3 Proteomic data analysis

**Fig. 2:**
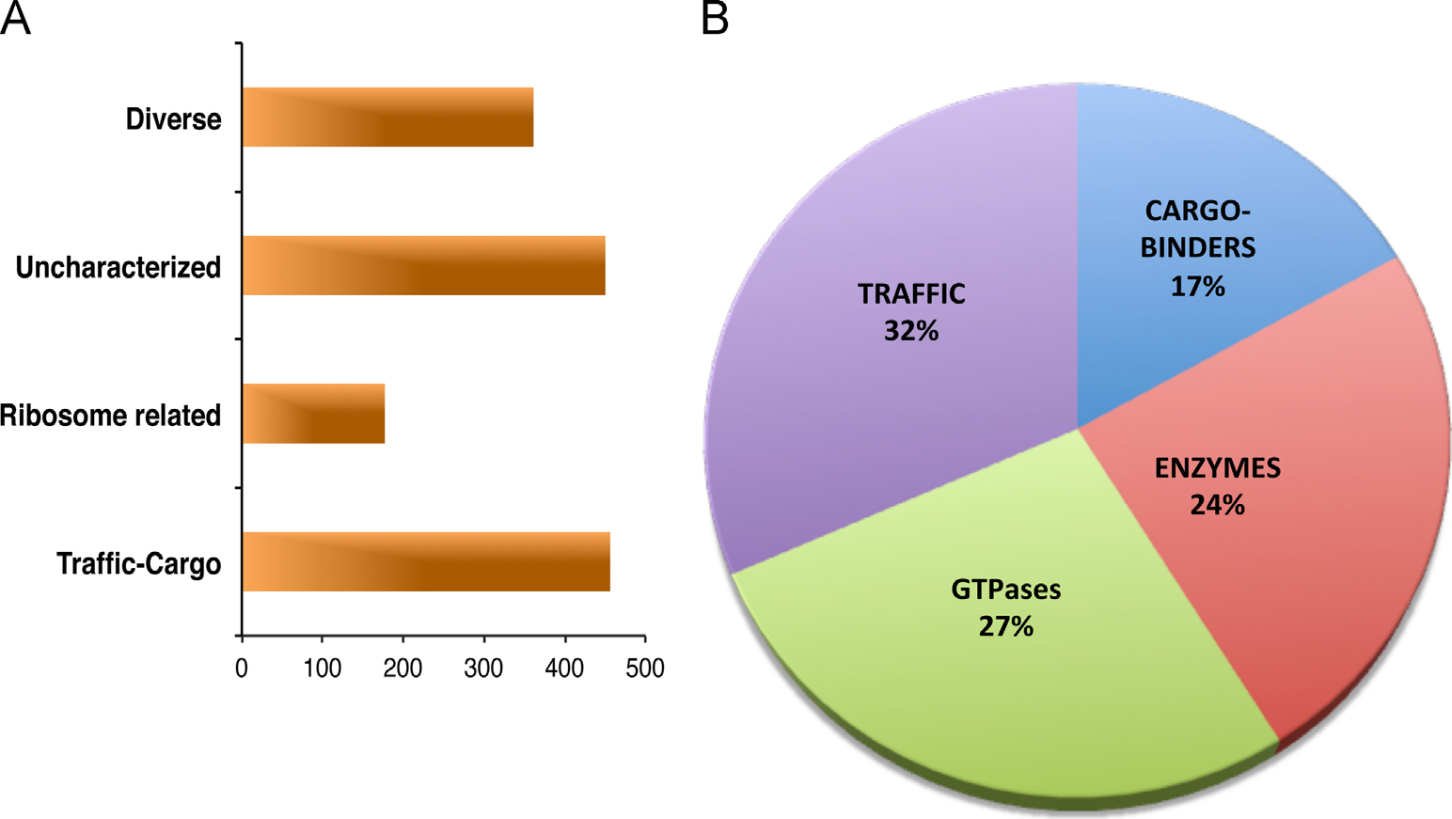
LC MS/MS identified proteins indexed in their corresponding categories. (A) Categories present from proteins identified in the isolated internal membrane fraction. (B) Percentage of proteins related to endomembrane compartments. (Taken from Perdomo et al. [1]).

Data analysis was performed using Thermo Proteome Discoverer software suite (version 1.4). For the search engine SEQUEST, the peptide precursor mass tolerance was set to 10 ppm, and fragment ion mass tolerance was set to 0.6 Da. Carbamidomethylation on cysteine residues was used as fixed modification, and oxidation of methionine along with N-terminal acetylation was used as variable modifications. Spectra were queried against the *E. histolytica* uniProt database. In order to improve the rate of peptide identifications percolator node in proteome discoverer was utilized with the false discovery rate (FDR) set to 1% for peptide and protein identifications. The identified protein list was further arranged in protein groups based on common peptide matches. For a comparative analysis of all the identified peptide and protein lists among the three biological replicates (the three internal membrane samples) a common merger table was generated and provided in Supplemental material 1, Table 1 (Sheet 1). All the individually sample specific protein groups and their corresponding peptide list are also presented in Table 1 (Sheets 2–6). A summary of the identified proteins and their corresponding functional category is represented in Fig. 2. For detail analysis, each category group of proteins is listed in Supplemental material 1, Table 2 (ER, Golgi apparatus, heat shock, TGN-ER retrograde transport, Endosomes and MVBs), Supplemental material 1, Table 3 (proteins with potential enzymatic activity associated to internal membranes), Supplemental material 1, Table 4 (GTPAses) and Supplemental material 1, Table 5 (possible cargo proteins). Proteins of unknown function present in the endomembrane fractions are listed in Supplemental material 1, Table 6 and of multiple functions are presented in Supplemental material 1, Table 7.

In order to determine the absolute abundance of different proteins within a single sample we used iBAQ feature of MaxQuant version 1.4.0.5 software using default search parameters [5,6]. The results of proteome discoverer and Maxquant searches were arranged together. The mass spectrometry proteomics data have been deposited to the open access library of ProteomeXchange Consortium (http://www.proteomexchange.org) via the PRIDE partner repository [2] with the dataset identifier PXD000770.

### 1.4 Bioinformatic analysis

Proteome discoverer annotation node, which is connected to ProteinCenter web based application, was used to download categorical GO database information in the form of biological process (BF), molecular function (MF), and cellular component (CC). Maxquant and Perseus were utilized for protein identification and assignment of Interpro, KEGG, Prosite annotations along with their iBAQ values. The iBAQ values were obtained by Maxquant software and are represented in.txt files representing protein and peptide identification results of the endomembrane enriched fractions.

**Fig. 3:**
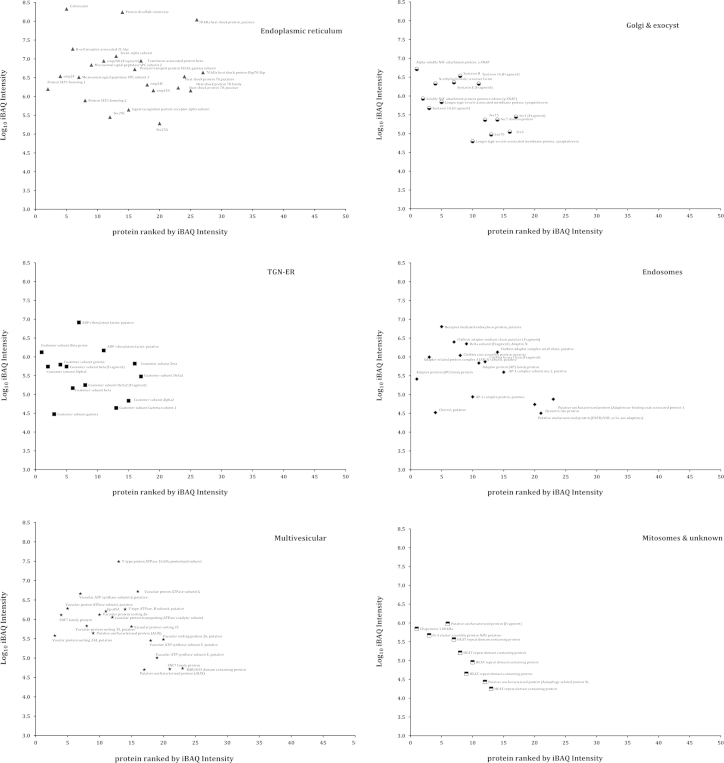
Proteins identified by LC/MS/MS with measurable iBAQ values corresponding to the endomembrane-trafficking system. After applying the iBAQ algorithm on the three raw files containing 1531 proteins, 1015 proteins had measurable iBAQ values and were separated into categories related to the ER, Golgi apparatus, TGN-ER, endosomes, MVBs, mitosomes and unknown. The iBAQ values varied over 5 orders of magnitude with respect to the most abundant and least abundant proteins. iBAQ analysis showed calreticulin to be the most abundant protein among all.

**Fig. 4:**
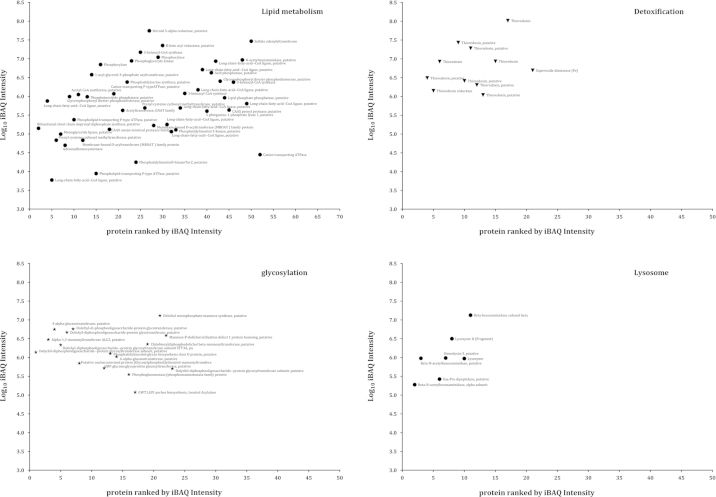
Proteins identified by LC/MS/MS with measurable iBAQ values corresponding to the enzymatic activities. After applying the iBAQ algorithm on the three raw files containing 1531 proteins, 1015 proteins had measurable iBAQ values and were separated into categories related to lipid metabolism, glycosylation, detoxification and lysosome. The iBAQ values varied over 5 orders of magnitude with respect to the most abundant and least abundant proteins.

**Fig. 5:**
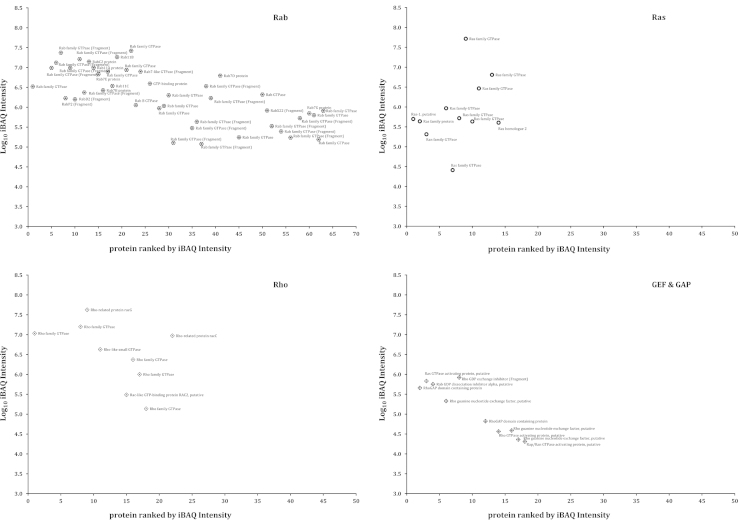
Proteins identified by LC/MS/MS with measurable iBAQ values corresponding to the G-ATPases family. After applying the iBAQ algorithm on the three raw files containing 1531 proteins, 1015 proteins had measurable iBAQ values and were separated into categories related to Rab, Ras, Rho, GEF and GAP. The iBAQ values varied over 5 orders of magnitude with respect to the most abundant and least abundant proteins.

A list showing dynamic range of internal membrane proteome of *E. histolytica* are represented as iBAQ values in Figs. 3–5.

**Fig. 6:**
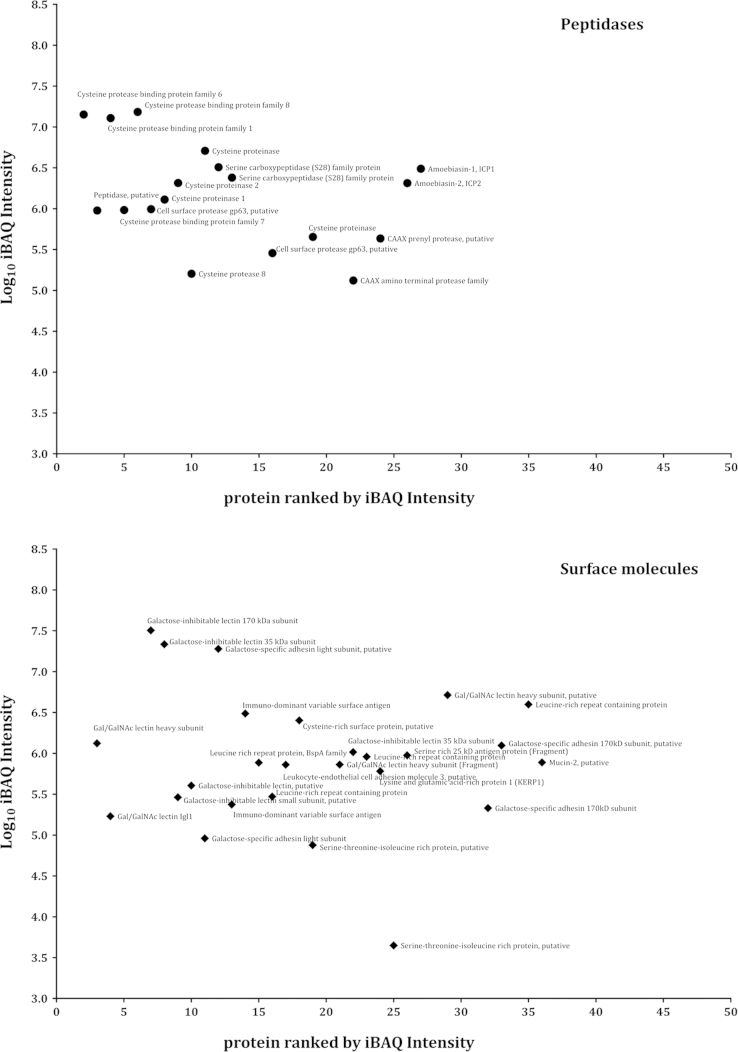

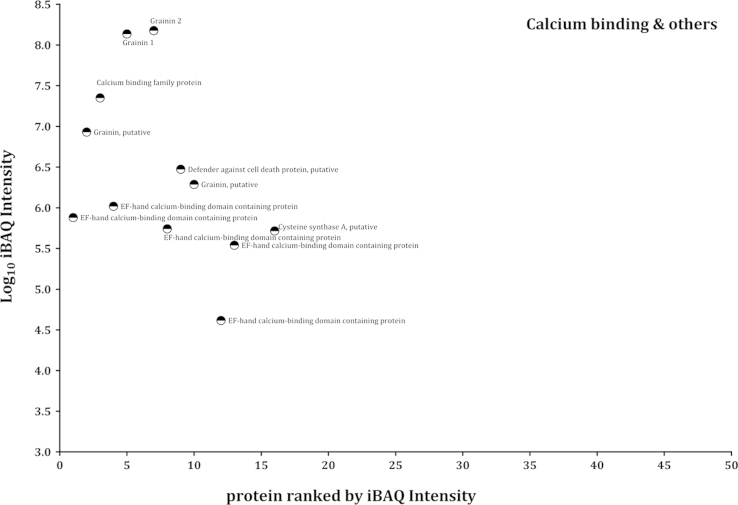
Proteins identified by LC/MS/MS with measurable iBAQ values corresponding to potential cargoes. After applying the iBAQ algorithm on the three raw files containing 1531 proteins, 1015 proteins had measurable iBAQ values and were separated into categories related to proteins that could be transported such as peptidases, surface molecules and calcium binding. The iBAQ values varied over 5 orders of magnitude with respect to the most abundant and least abundant proteins.

Maxquant results and analysis are found as a folder in Supplemental material 2 (Fig. 6).

## Conflict of interests

The authors declare that they have no competing interests.

## Figures and Tables

**Fig. 1:**
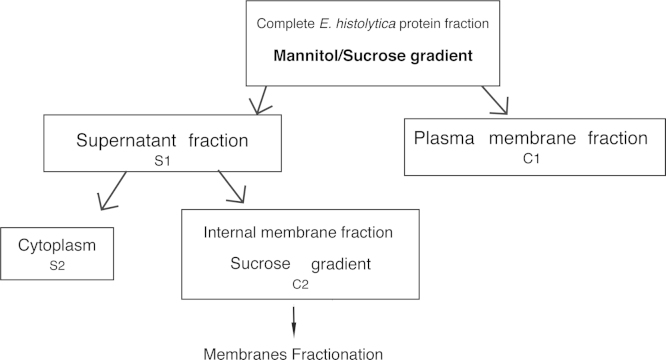
Scheme of the procedure used for separation of endomembrane enriched fraction. *Entamoeba histolytica* subcellular fraction separation was performed as described before [4] from a trophozoite pellet corresponding to 2×10^8^ cells. The final yield of internal membrane proteins was of 50 μg/µl.
